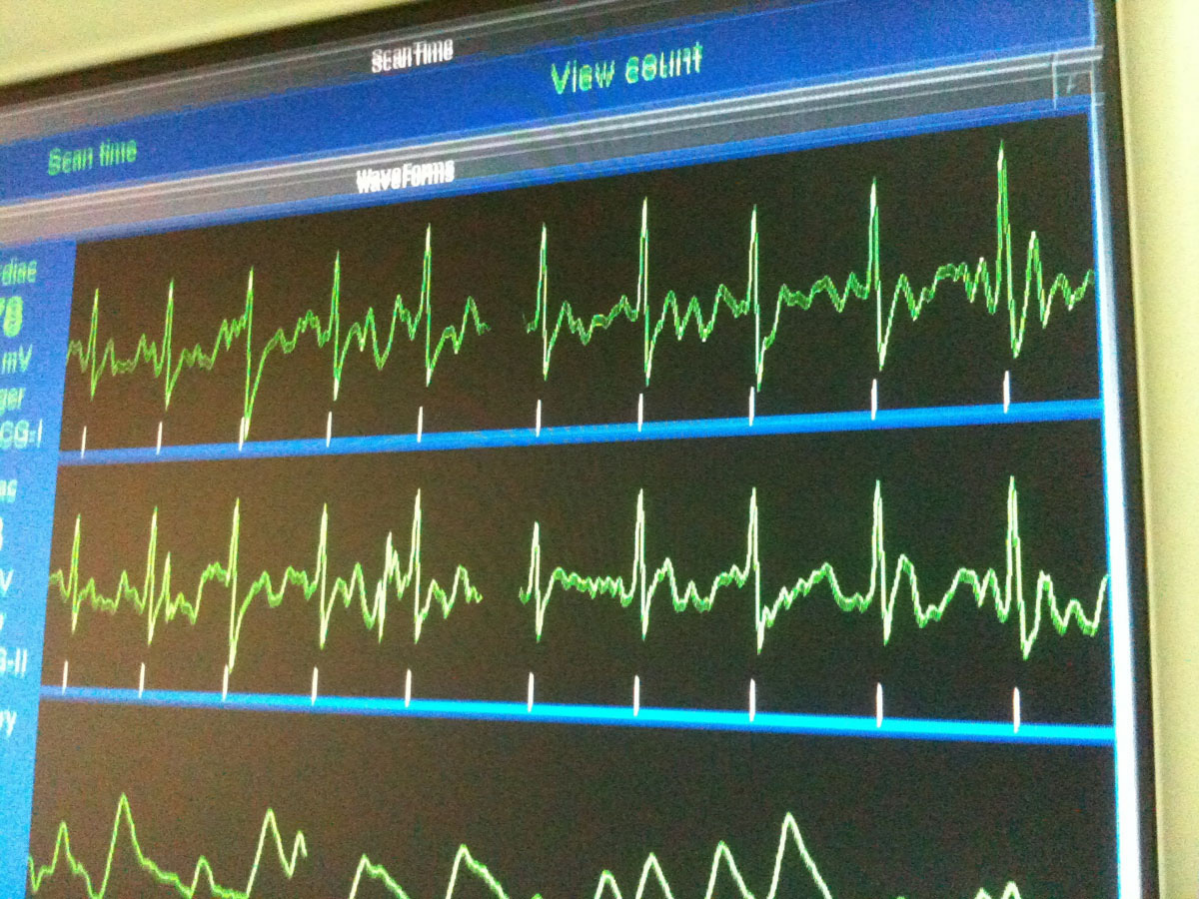# Improve of arrhythmogenic right ventricular cardiomyopathy/dysplasia evaluation by magnetic resonance using ventricular arrhythmia suppression with intravenous lidocaine

**DOI:** 10.1186/1532-429X-14-S1-O25

**Published:** 2012-02-01

**Authors:** Afonso A Shiozaki, Karen A Koga, Andressa Giordani, Taisa V Lorencete, Guilherme C Nogueira

**Affiliations:** 1 Cardiovascular Magnetic Resonance Sector, Instituto Maringá de Imagem (Maringá Imaging Institute), maringa - Parana, Brazil; 2 Fleury, São Paulo, Brazil

## Background

New recommendations from task force criteria for the diagnosis of arrhythmogenic right ventricular cardiomyopathy/dysplasia (ARVC/D) include regional right ventricle (RV) akinesia or dyskinesia or dyssynchronous RV contraction associated to RV dilatation or RV dysfunction evaluated by magnetic resonance imaging (MRI) as major diagnostic criteria. However the frequent presentation of ventricular arrhythmias during the acquisition of MRI results in significant limitations in the evaluation of these images.

Lidocaine has been used clinically in the suppression of ventricular arrhythmias. Objetctive: we used lidocaine to attempt ventricular arrhythmias suppression during MRI acquisition in order to improve the quality images to assessment of function and focal segmental contractility of RV in the evaluation of patients with ARVC/D suspected.

## Methods

We included 20 patients with ARVC/D suspected submitted to MRI presenting frequent ventricular arrhythmias (mostly ventricular bigeminy and trigeminy) during the acquisition of MRI. First we performed the Stead State Free Precession (SSFP) to RV evaluation in the presence of ventricular arrhythmias and after we administrated 1mg/kg of intravenous lidocaine in 30s and repeated the SSFP images to RV re-evaluation.

Before and after the administration of lidocaine we registered the frequency of ventricular arrhythmias and analyze the image quality of global and regional RV function in (1 excelent, 2 good, 3 regular and 4 poor quality of images).

## Results

We observed significant reduction of ventricular arrhythmias on subsequent five minutes after administration of lidocaine with suppression of bigeminy and trigeminy in 75%. We observed significant better quality of images in 80%. Moreover none relevant side effect due lidocaine was observed.

## Conclusions

In evaluation of ARVC/D by MRI, the use of lidocaine can improve the image quality in patients presenting ventricular arrhythmias during the acquisition of exam.

## Funding

None.

**Figure 1:**
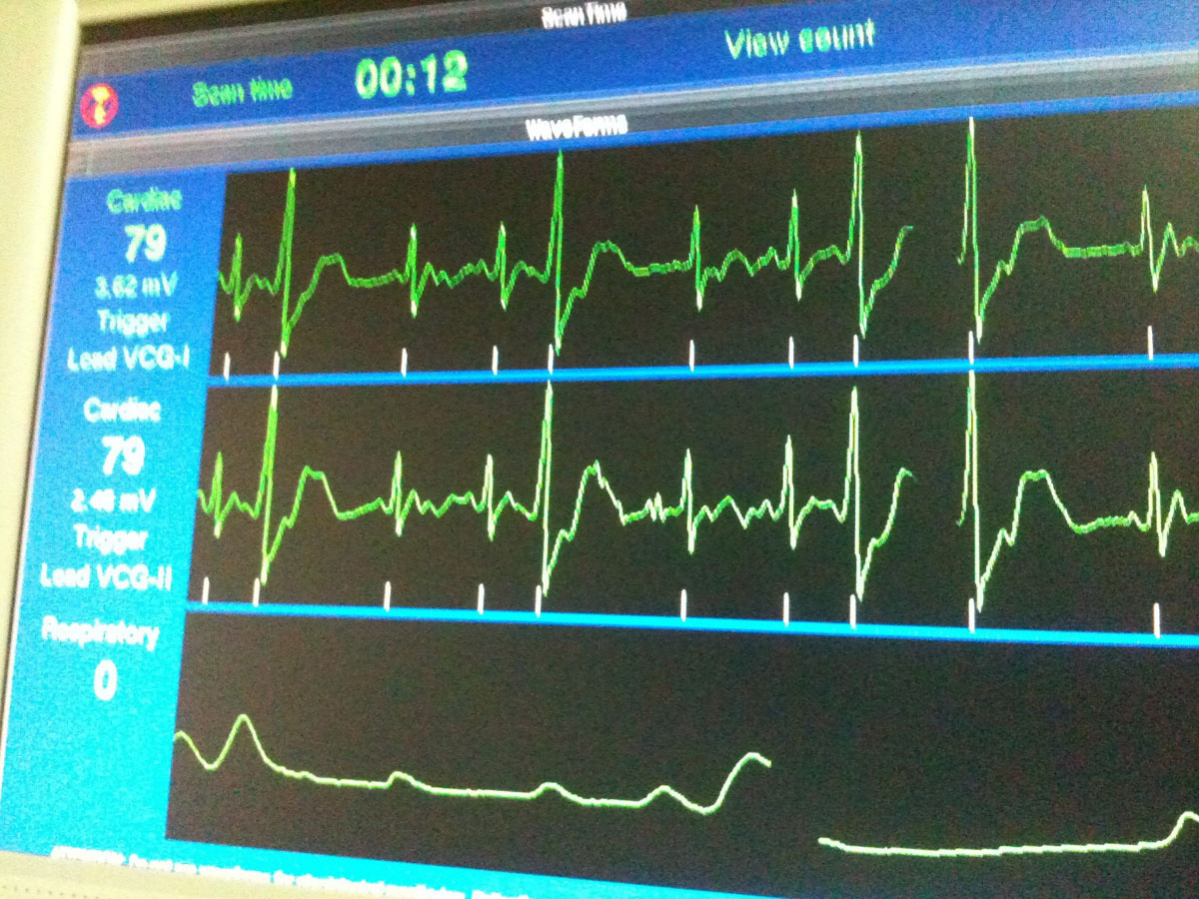


**Figure 2:**